# Cyclic workflow to improve implementation of learning points from morbidity and mortality meetings

**DOI:** 10.1186/s12913-022-08639-2

**Published:** 2022-10-25

**Authors:** B.J. Myren, J.A. de Hullu, J.J. Koksma, M.E. Gelderblom, R.P.M.G. Hermens, P.L.M. Zusterzeel

**Affiliations:** 1 Department of Obstetrics and Gynaecology, Radboudumc, Nijmegen, The Netherlands; 2 Health Academy, Radboudumc, Nijmegen, The Netherlands; 3 IQ Healthcare, Radboudumc, Nijmegen, The Netherlands

**Keywords:** Healthcare quality, Organizational change, Group meeting, Patient participation, Workflow, Learning, Complications

## Abstract

**Background:**

Morbidity and mortality meetings (M&MMs) are organized in most hospital departments with an educational purpose to learn from adverse events (AEs) to improve patient care. M&MMs often lack effectiveness due to unsuccessful systematic follow-up of areas of improvement. This can have an effect on improving patient safety and care. Therefore, a new strategy that focuses on implementing areas of improvement into daily practice is necessary. The study aim is to see if we could improve the implementation of meeting outcomes from the M&MM by using a cyclic workflow, and which factors are important to achieve its implementation.

**Methods:**

This prospective study took place at the department of gynecologic oncology of a university hospital. Research was conducted with a participatory action research (PAR) approach using 10 consecutive M&MMs in 2019 and 2020. The cyclical workflow consisted of an action list based on the PDCA-cycle, a check of the implementation of areas for improvement at the next M&MM and regular monitoring of tasks. Each M&MM was observed and each professional with an assigned task was interviewed and gave their informed consent. Thematic content analysis was performed with the program Atlas.ti 8.4.20.

**Results:**

Out of the 39 tasks that resulted from 10 M&MMs, 37 (94.8%) followed all the steps in the PDCA-cycle and were implemented. In total, 16 interviews were conducted with consultants, nurses, registrars and residents. Five main factors were important to achieve follow-up of areas for improvement: organizational culture, motivation, commitment, communication to mobilize employees and skills. Repetition of the cyclic workflow at the M&MM and an external person who reminded professionals of their assigned task(s) was important to change habits and motivate professionals.

**Conclusion:**

Cyclical tools can support the implementation of areas for improvement to optimize the M&MM. A M&MM with an organizational culture where attendees can discuss openly and freely may motivate attendees to take on tasks successfully. A positive stimulant to reach commitment of professionals is team participation. Integrating new habits of reflection may lead to a deeper level of learning from the PDCA-cycle and of the M&MM. Creating a learning environment outside of the M&MM may support professionals to take on actions and engage in improvement practices. Future research may focus on including a comparative analysis to show a success rate of the implementation of learning points from the M&MM more clearly.

## Introduction

Morbidity and mortality meetings (M&MMs) are important to improve patient safety and surgical quality of care. [1, 2] Having an effect on medical practice is in particular important for M&MMs, which are organized in most hospital departments with an educational purpose to learn from adverse events (AEs) to improve patient care. [3, 4] However, although areas for improvement result from the M&MM, the desired expectation of practice change or systematic follow-up of areas for improvement lack effectiveness. [5–9] The learning points resulting from the M&MM are not succesfully implemented in daily practice. In order to improve the methods of the M&MM and support the implementation of areas for improvement in daily practice, professionals need to change their routine behavior. [10] Therefore, an improvement strategy that strengthens the systematic follow-up of areas for improvement may need to focus on active engagement of professionals. [11].

Next to the engagement of professionals, research outlines strategies for effective meetings with tangible results. [12–14] These include, having routine items, such as closing the meeting with a clear delegation of follow-up points and using an agenda with recurring actions. [12–15] A popular cyclical workflow that includes both elements, is the Plan-Do-Check-Act (PDCA)-cycle. This PDCA-cycle is a method to continuously improve quality of processes. It systematically follows the process of making a time-based improvement plan, recognizing and analyzing problems, to finally follow the steps leading to improvement in practice. [16] Even though this workflow contributes to a cycle of learning that includes elements of reflexivity [17, 18], more research is needed to understand the effectiveness and sustainability of the PDCA-cycle. [19, 20] Quality improvement should itself be viewed as a learning process for professionals and the organization. [21] Therefore, the PDCA-cycle may function as a way to optimize the working environment as a learning environment by increasing the frequency of reflecting and acting by professionals. [22] The follow-up of areas for improvement from the M&MM may depend on the professional’s attitude towards including these tasks in practice. Moreover, behavioral change is key to support the successful uptake of an improvement strategy which requires learning processes. [23, 24] Therefore a work environment, or organization, should stimulate that type of learning. Especially in a healthcare context where professionals have routine behavior and set ways of working. Although there are few examples of successful implementation of the PDCA-cycle at M&MMs, this has not become common practice in most hospital departments. [25].

In 2016 the department of Gynecologic oncology of an academic Dutch hospital in the Netherlands successfully implemented patient participation at the M&MM as standard care. [7, 26–27] Our research, as well as other studies, showed that patient participation at similar meetings result in different and new perspectives and improves the meeting. [7, 26, 28–30] For example, well prepared meetings because it created an additional urgency to provide evidence based arguments and new insights in comprehensible language for the patient, and diverse learning points in the field of collaboration and communication. In our department the professionals experienced commitment to communicate a written report of the status of the meeting outcomes to the patient after three months. However, learning points from the M&MM were not always implemented in practice and specific practical tools to record and enable healthcare professionals to engage with the follow-up of meeting outcomes were lacking. Therefore, we needed a strategy that focuses on systematic follow-up of areas for improvement from the M&MM with patient participation. Practical tools were developed with elements of the PDCA-cycle in order to include a cyclic workflow. Due to the importance of changing professionals’ routine behavior, this study also focuses on factors that may explain the underlying processes that positively or negatively affect the implementation of a cyclic workflow. This can be studied by observing closely what works and what does not, and by encouraging participants to take action. [31, 32] These processes should be better understood to provide generalizable advice on the level of organizational culture, motivational drivers and group interaction at the M&MM. [33].

The aim of this prospective study was to improve the implementation of meeting outcomes from the M&MM by using a cyclic workflow, and which factors are important to achieve its implementation.

## Materials and methods

### Research setting & improvement strategy

**Fig. 1:**
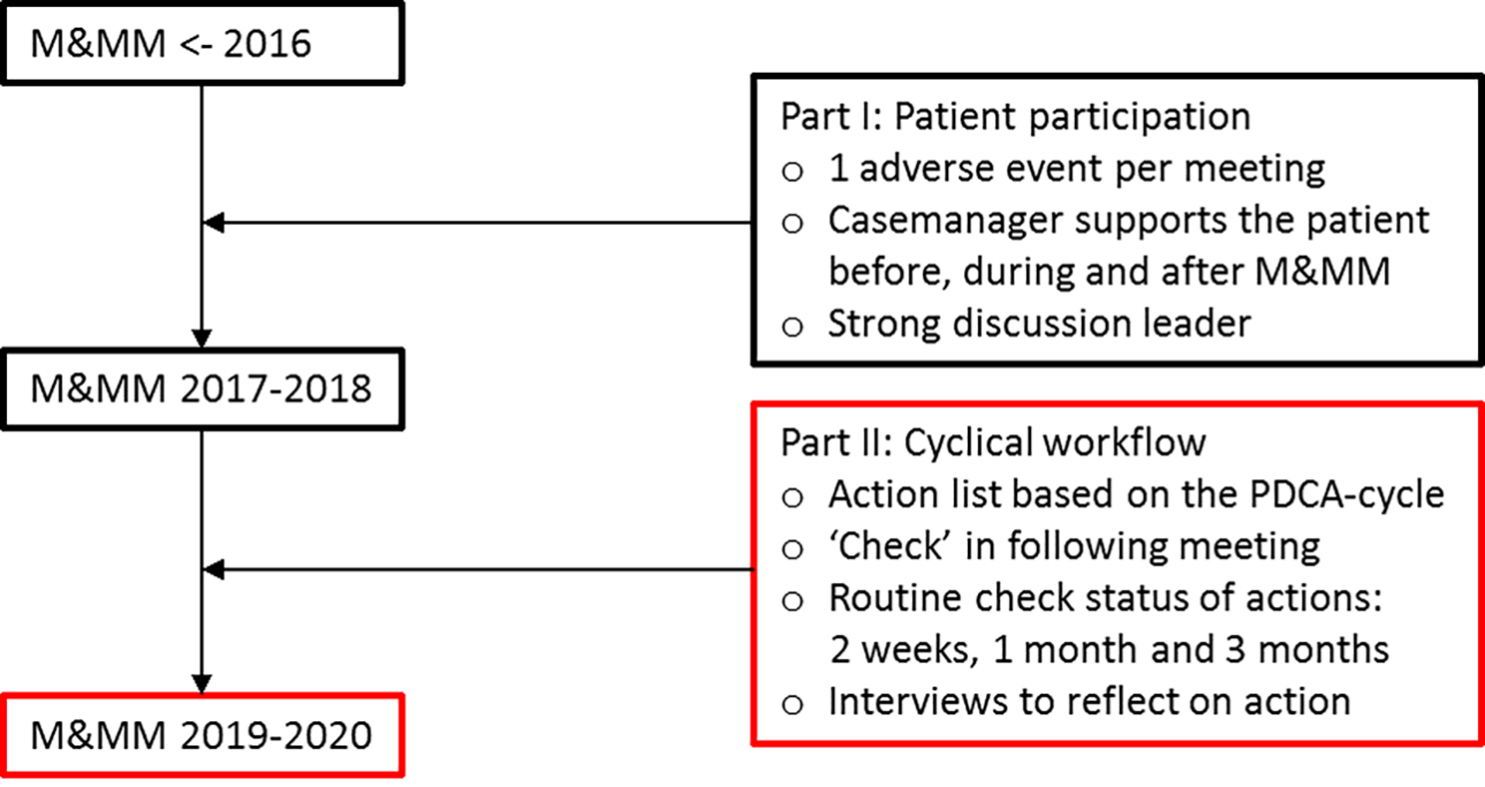
Elements of improvement strategy part I and II implemented between 2016 and 2020

The research took place at the monthly M&MMs at the department of gynecology of a university hospital in the Netherlands. Figure 1 outlines improvement strategies part I & II implemented to improve the M&MM since 2016. The first improvement strategy patient participation changed the meeting structure by inviting all the involved participants. [3] The goal of the traditional M&MM did not change with patient participation: one AE is discussed with the aim to learn from what happened and to improve practice. However, some adjustments were made: professionals used comprehensive language, the goal of the meeting was explained prior to every meeting, and all attendees were introduced to the patient (and companion). The patient was invited to bring a companion, such as their partner, a family member or a close friend who could also share their experience. During the M&MM the patient (and companion) had time to share their experience, provide feedback and join the discussion. The chair was an independent consultant from the department, experienced in chairing M&MMs. The presentation was conducted by a fellow or senior registrar involved in the case, supervised by the consultant. Regular attendees were the patient and a companion, gynecological consultants, registrars, residents, research nurse and casemanager from the department. Occasionally consultants from other departments (anesthesiology, surgery, urology, etc.) and nurses from the ward attended as well, depending on the case .

The second improvement strategy cyclical workflow was developed in 2019, and included PDCA-cycles. The practical tools were co-designed with healthcare professionals familiar with common barriers of implementing areas for improvement into daily practice, members of the hospital emergency management committee and the executive researcher (BM). The tools of the cyclical workflow were based on existing PDCA tools used by the hospital emergency management committee and current practice of the M&MM. These included an action list based on the PDCA-cycle (Table 1), personal contact (BM) to follow-up on the tasks after two weeks, one month and 3 months (when necessary); Reserving the last 15 min for reflecting on tasks from the previous meeting(s) was a new structured item of each M&MM with patient participation. Successfully executed tasks followed all the steps in the PDCA-cycle as shown in Table 1 and were implemented accordingly.

**Table 1:** Primary columns of the action list based on the PDCA-cycle

			PLAN		DO		CHECK	ACT
Nr	Outcome of M&MM	Professional with assigned task (name/ function)	Desired result (goal setting)	Steps to achieve goal setting	*Start date improvement activity*	*Desired date to finish improvement activity*	Check whether task is completed (date /how)	Additional measures and/ or actions

### Research design

#### Participatory Action Research (PAR) & participants

Qualitative research methods were used to evaluate the factors involving the successful implementation of the practical tools to understand ‘what works, why and under which circumstances’. [34] Participatory action research (PAR) is a methodology that can involve the researcher as a participant in the research context to collect data. [34] In our study the researcher attended each M&MM, created each action list and communicated with healthcare professionals on the status of their tasks. This way the PAR approach stimulated an exchange between the participants in the study and the executive researcher. [35–36] It provided opportunities for continuous attention to observe and recognize patterns of behavior over time. [36–38] In addition, interim results of the study were shared during the implementation of the cyclical workflow with research team members who attended the M&MM, while collecting data.

### Data collection

Qualitative data was collected from 10 M&MMs with patient participation in the period of 2019–2020.

#### Interviews

Semi structured interviews were held in Dutch by the executive female researcher (BM) with all professionals who were assigned to a task on the action list. The researcher was familiar with professionals who attended the M&MM regularly. One of the inclusion criteria to participate in the research was to have a task assigned at the end of the M&MM. Professionals were invited to join the research via e-mail. In total, 16 interviews were conducted with consultants, nurses, registrars and residents and lasted between 12 and 32 min. The open interview questions focused on practicalities, possible difficulties in finalizing the task and why it was (un)successful. In addition, the interviews inquired about the impact of using the practical tools implemented at the M&MM, their motivation to finish the task, whether they shared the outcomes with their colleagues and whether it impacted their view on patient-centered care. All interviews were held via telephone, recorded and transcribed verbatim.

#### Observations

Each consecutive M&MM was observed by the executive researcher (BM). These observations focused on the formulation of action points during the meeting, whether attendees took on tasks and the division of tasks. A task was successfully executed when the professional finalized and implemented each step of the PDCA-cycle described on the action list. The researcher used fieldnotes during the observations, which were extensively written down after each M&MM. E-mails that involved actions were also included as data.

### Data analysis

The transcribed interviews were analyzed in the program Atlas.ti (version 8.4.20, Atlas.ti Scientific.

Software Development GmbH; Berlin, Germany) by two coders (BM, MG). The content analysis method and elements of narrative analysis were used, such as coding larger blocks of text to better take the professionals’ full story into account. [39] In the first round, both coders used open coding in three different transcripts. Based on these codes different broad categories were clustered. In the second round the transcripts from the first round were coded again with three additional transcripts. After each round both coders discussed the categories to detect missing topics, or the relationship between the categories. The coders discussed upon agreement with quotes or parts of the transcripts to support the arguments. The authors (PZ, RH, JH, JK) provided feedback on the code tree and came to agreement in a meeting before the third round of coding started. In the third round the other transcripts were coded and the theoretical perspective from literature was added to define the themes found in the content analysis. The observations were used to contextualize the interview transcripts during the analysis of the interviews and to detect behavioral changes. A rating of importance was given to the factors. This was based on both interviews and observational data that showed which of the factors eventually led to following all the steps in the PDCA-cycle and successful implementation.

### Ethical considerations

All methods were carried out in accordance with relevant guidelines and regulations under Ethics approval and consent to participate. This research was approved by the local Medical Ethical Committee of the hospital (‘CMO Regio Arnhem-Nijmegen’) case 2020–6142. Prior to the interview professionals received information concerning the research, gave their informed consent and knew the researcher was involved in different research focusing on improving the M&MM. The attendees were made aware of the presence of a researcher observing during the M&MMs. The interview recordings were deleted after transferring them to a computer with folders protected by a digital key. The COREQ checklist was used during the research to adhere to criteria for reporting qualitative research. [40]

## Results

We found that improvement strategy cyclic workflow aids in the follow-up of areas for improvement at M&MMs based on the analysis of 10 meetings. Five main factors were important in the uptake and implementation of tasks. In the following section we present six successfully implemented PDCA-cycles, and the five factors that are important for the implementation of the improvement strategy.

### Actions and successful examples

The 10 M&MMs resulted in action lists with 2 to 5 actions per meeting. 37 of the 39 tasks (94.8%) followed all the steps in the PDCA-cycle and were implemented. Each task was assigned to a professional. Additional steps were described and added to the action list in the last column when the tasks were checked, usually at the following M&MM. Two out of the 39 tasks were not finalized or completed. These tasks were assigned to professionals from a different, external department and included follow up of research on a procedure. Paragraph 3.2.2 explains that it took more time and attention to motivate professionals from external departments to finalize tasks. Table 2 shows six successfully finalized tasks with a description of the AE that was discussed during the M&MM.

**Table 2:** The action list tool with six successfully implemented tasks

#	Adverse event (AE)	Outcome of the morbidity and mortality meeting (M&MM)	PLAN: Desired result (goal setting)	DO: Steps to achieve goal setting	CHECK/ ACT: Check whether goal is achieved and whether additional steps are needed
1.	2020, January Haemorrhage (blood loss > 500ml) after deep excision	Insufficient knowledge about hemostasis material in colposcopy room.	Inventory of (available) options for haemostatic material (websearch) after a leep excision.	Liquid silver nitrate and Surgicel© as standard absorbable hemostats in the colposcopy room. All team members are aware of which materials are present and where its stored in the colposcopy room.	Lecture held on absorbable hemostats. Liquid silver nitrate is no longer available.
2.	2020, February Recurrent urinary tract infection due to urinary retentions after removing the bladder catheter too soon following a Wertheim-Meigs procedure.	Make bladder scans required after Wertheim-Meigs procedure when catheter is removed as urine retention occurs more often. If necessary, long term catheter à demeure, self-catherization are options.	Standard of care protocol used at the ward which bladder retentions are acceptable after a specific procedure	Healthcare professional assigned to the action will check and modify the protocol if necessary.	There is a standard protocol at the inpatient department after surgical procedures of urology, surgery and gynaecology. Protocol does not need to be modified. Additional action: Everyone is aware of this hospital wide protocol.
3.	2020a, June Wound dehiscence (“space belly”).	The suture used for closing the fascia was too short. Two sutures were tied together, leading to a weak spot.	Based on new advice - long polydioxanone suture (PDS) barrel (300 cm) is ordered.	Order long PDS barrel.	The PDS barrel is ordered and since July 2020 both short PDS barrel (120 cm) and long barrel (300 cm) are available.
4.	2020, July Excessive CO2 accumulation during a laparoscopic procedure.	Clear communication during surgery about peri-operative issues between anesthesiology team and operative team. In patients with higher BMI, the use of longer trocars is necessary to prevent CO2 leakage subperitoneal.	If necessary, introduce additional time-out during OR in case of impending complications. Order longer trocars.	Repeat the outcome at the following M&M meeting. Order longer trocars. Create awareness of the risk of CO2 accumulation during surgery.	Actions are accomplished, and discussed again at the following meeting.
5.	2020, August Overbalanced liquid intake postoperatively.	The liquid balance was not documented.	During each bedside rounds the liquid balance is documented (input and output within a 24-hour period in millimeters).	Organize education on liquid balance for registrars and nurses and at the inpatient ward. State liquid balance in the electronic patient file at every bedside round.	Additional education for registrars and nurses on the overbalanced liquid intake policy postoperatively took place.
6.	2020, September Wound infection.	High risk of infection after inguinal wound. Particularly in patients with obesity. Research of other products that may aid in wound repair.	Start flushing the wound postoperatively with povidone iodine solution. Communicate with infection prevention/ hospital hygienist to determine how to reduce the risk of wound infection with these procedures. -> Evaluate the outcome of the use of povidone iodine solution at the end of surgery in relation to wound infections	Each staff member will record in the OR report whether the wound was flushed postoperatively for 6 months. Schedule appointment with hospital hygienist.	Each staff member is aware that whether or not the wound was flushed with povidone iodine solution needs to be stated in the patient’s operating report. Contact has been made with hospital hygienist at a later stage (Due to COVID-19 this was postponed). After 6 months the use of povidone iodine solution was evaluated (data from operating reports).

### Relevant factors for successful implementation of areas for improvement

**Fig. 2:**
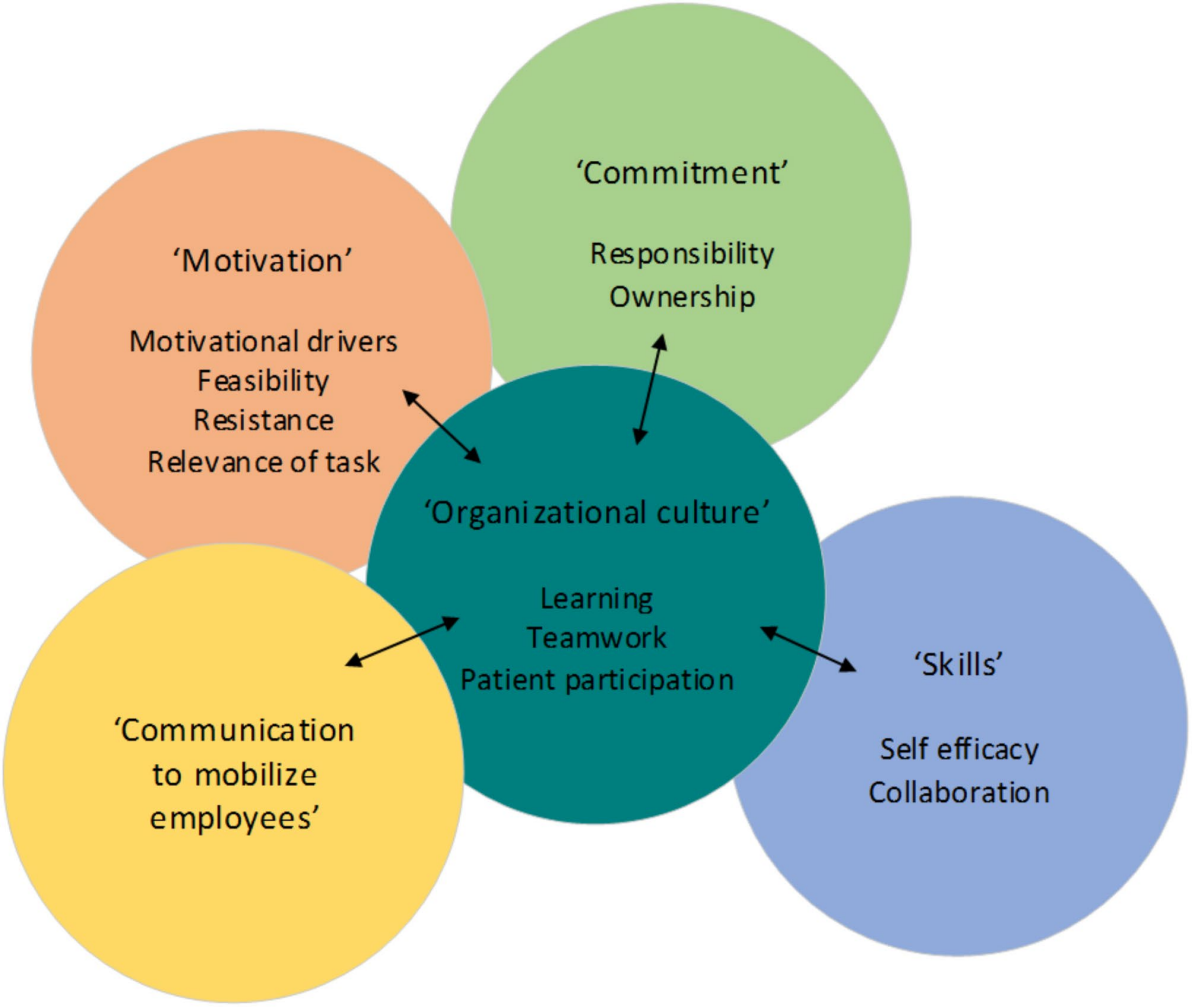
Five factors relevant for the successful implementation of the improvement strategy

Analysis showed five main factors explaining underlying reasons of why professionals included their assigned task in their routine behavior: organizational culture, motivation, communication to mobilize employees, commitment and skills (see Fig. 2). The most important factor was organizational culture that influenced the four other factors. Motivation was the second most important factor that influenced three other factors. All professionals who received a task at the M&MM accepted the invitation to join the research and were interviewed.

Figure 2 shows the five main factors and sub themes. These five factors will be illustrated by a successfully executed task from Table 2: ‘overbalanced liquid intake postoperatively’.

#### Organizational culture

Professionals explained a supportive culture as an environment where; outcomes were discussed openly without judgement, they can admit to mistakes and receive support when a task was not yet successfully finalized. For example, at the M&MM concerning the overbalanced liquid intake postoperatively there was an open discussion that led to a clear division of tasks (see Table 2, case number 5.). The nursing staff as well as a consultant together with a registrar who did not attend the M&MM, were assigned with a task. In general, the implementation of areas for improvement was effective when professionals could easily collaborate with colleagues, when there was a clear division of work-tasks and when professionals could use their own creativity to finalize the task. The latter was especially important when the task was not part of the daily responsibilities of the professional. The action list and the continuous communication with the researcher were accepted quickly by participants because experienced professionals responsible for the M&MM introduced the improvement strategy. Professionals explained in the interviews that the culture at the M&MM, and outside of the M&MM, provided an environment that supported behavior change and also motivated professionals to actively engage with their task.

*‘For me it is no problem to say hey guys maybe I did not do this in the right way. … Because when you discuss this with people [colleagues] they will support you and look into it to see what it is you did.*’ (#8, registrar gynaecological oncology).

#### Motivation

In the example of the overbalanced liquid intake postoperatively, nurses were motivated because they were aware of the importance of their task, they felt responsible and were capable of executing the task. The registrar who did not attend the M&MM was not motivated at first. Although it was the registrar’s responsibility to organize monthly education, this caused resistance as she only received a short e-mail, was unaware of the AE and unfamiliar with the case of that patient.

In general, motivation was explained by the motivational drivers of professionals (reasons why professionals feel motivated), when a task on the action list was feasible, when the action suited the professional’s role and responsibilities and when it was seen as relevant (see Table 3). Moreover, when an action suited the professional’s role, the professional felt a sense of ownership that enhanced their motivation. Professionals experienced resistance when they did not feel taken seriously, did not have enough knowledge about the AE, or when they were not present at the M&MM. The visibility of practice change by sharing the result of a succesfully executed task was an important motivational driver for most professionals. Therefore, the action list needed to be shared with the team and stored in an accessible location.

*‘Look, everything that can improve the quality of care, that is something we should do. And that should be disseminated as well. Because there are also people who say ‘I did not hear about it afterwards and what is the situation right now’. Then you can say what we agreed upon is here on the drive and this was sent around.’* (#6, consultant gynaecological oncology).

External factors could positively or negatively influence the motivation of professionals. Professionals explained that they became more motivated when the researcher used e-correspondence to check the feasibility and status of the task prior to the following M&MM. This was especially important for professionals from other departments, because they did not attend the following M&MM where tasks were checked and reflected upon. The additional personal contact was crucial at the start of the intervention, because professionals needed to get acquainted with, and reminded of, their assigned task on the action list.

**Table 3:** Overview of motivational drivers, resistance and general advise for underlying reasons of (un)successful use of the improvement strategy

Motivational driver	Resistance found during research	General advise
A sense of ownership	• Professionals who were not present during the M&MM and received a task afterwards • Professional who were made responsible for a task without giving consent	• Explain the assigned task via phone call or an explanatory email • Explain clearly when a task is part of someone’s roles and responsibilities
Clear task description with deadline and an environment that promotes independency	• Unclear task/ not feasible • Professionals who lacked the skill to be creative and autonomous in finishing tasks • The person who described the tasks on the action list lacked the skill to do this consistently and clearly	Describe the task SMART (Specific, Measurable, Achievable, Realistic, Time-related)
A sense of urgency and relevancy of the task	Professionals who received too many emails; emails may be overseen	Repeat tasks in other (weekly) meetings
Visibility of the status of the tasks	Professionals who did not see or find the status of the task, and/or the task is unclearly written	• Make the action list available on a shared location • Provide regular (short) updates by e-mail with the status of the tasks
Multidisciplinary M&MM with new perspectives and inter-departmental support to execute tasks	Professionals from other departments did not feel motivated to finalize the task	• Organize regular M&MMs with other departments • Create extra contact moments with professionals from other departments about their tasks • Make someone from your own department responsible for the finalization of a task by another department
Visibility of practice change when tasks are executed	It was unclear whether the task influenced or changed daily clinical practice	Share task with colleagues and invite colleagues to support in completing the task
Improvement of the quality of care (described as a feeling)	Professionals who received a task that was unrelated to their daily responsibilities or work-role	The tasks should fit and align with the daily work-role of the person with a task assignment

#### Communication to mobilize employees

In the example of the overbalanced liquid intake postoperatively, nurses received an e-mail by the executive research (BM) with an informal and relatable tone. These nurses attended the M&MM and were aware of the AE and its consequences. They were also able to mobilize their colleagues and inform them about the policy changes (their task). The registrar did not attend the M&MM and therefore needed to receive a different way of communicating her assigned task.

‘*So I think you should assign tasks to people who are part of the group of attendees. And then the task will be to mail person x [person who did not attend the meeting], and the one who receives the task is person y [person who attended the meeting]’*. (#10, registrar obstetrics)

This could also have been a phone call with additional information or a more elaborate e-mail. Receiving a task, or checking for the status of that task, required a tone of mutual respect especially when a professionals was unable to attend the M&MM. In general, communication about the content of the task was important to mobilize professionals to actively engage with their task. During the improvement strategy the communication about the tasks occurred during the M&MM when areas for improvement were formulated, and in outside of the M&MMs by the researcher. This was only possible when the overall (hierarchical) culture provided space for another person (here: researcher) to assign tasks.

*‘Yes, well I have seen your name more often so I know what you do related to your research and so on. So I did not think it was weird that this questions came from you. No, definitely not.’* (#12, nurse gynaecological oncology).

In addition, it is important to have experience in how to clearly formulate and write tasks on the action list. We found that writing down tasks clear for all professionals required a specific skillset that includes medical knowledge. The executive researcher lacked medical knowledge and was therefore unable to describe some tasks.

#### Commitment

Our results showed that commitment went beyond motivation. This meant that a committed professional was always motivated to finalize his/her action. Both the nurses and the registrar were committed to finalize the task because it was part of their regular work description. Most professionals explained that they felt a sense of ownership and ultimate responsibility of the task, regardless whether they had enough time to follow-up on it.

*‘Yes, but either way, even when there is no problem, I feel ownership… So even when the task would have been assigned to someone else and it does not make sense. Even then, it could be possible that this person thinks I am responsible.’* (#11, consultant gynaecological oncology).

Due to the importance of commitment, the professional that takes on similar tasks in their daily work needed to be assigned to the task despite their full schedule. In order to make sure that a specific professional takes on this task, it required a professional, next to the researcher, knowledgeable of the different roles and responsibilities of each member in the team of professionals.

#### Skills

In general, self-efficacy was explained by a culture that stimulated professionals to start immediately on their task, plan necessary activities, be creative in finding solutions to execute the task and propose follow-up actions. In the example of the overbalanced liquid intake postoperatively, both the nurses and the registrar were able to finalize their task and collaborate. They planned the necessary activities to change the liquid balance policy and used their own creativity to organize the education. The organizational culture in the team may have supported them in using these skills.

*‘Most of the time my colleagues in gynaecology respond well to my feedback. Ofcourse, with a degree of exceptions.’* (#13, senior registrar gynaecological oncology).

Overall the action list supported professionals in their self-efficacy, because this provided a clear overview of tasks and deadlines. Professionals who were aware of the knowledge, network and daily work-tasks of other colleagues were better equipped to collaborate and execute the task on time.

‘*With these types of protocols, each nurse in our department has a specific focus such as pain, palliative care, or wound care. So first we need to meet with them to see what the protocol entails, whether everything is clear to them, how they ensure people use the protocol and whether they need to do something with the protocol related to the M&MM, or whether it was just an incident.’* (#6, consultant gynaecological oncology).

## Discussion

Areas for improvement resulting from the M&MM with patient participation can be successfully implemented into daily practice supported by a cyclic workflow and motivated professionals to take on and finalize tasks. Our study showed that 37 tasks (94.8%), of a total 39 tasks from 10 M&MMs, followed the PDCA-cycle and were successfully implemented. Five factors described the underlying reasons for the behavior of professionals while carrying out assigned tasks: organizational culture, motivation, communication to mobilize employees, commitment and skills.

We found that almost 95% of the tasks followed the PDCA-cycle and were implemented. This may indicate that the meetings were effective and the cyclic workflow created opportunities for successful meeting management in the context of M&MMs that include patient participation. [14,41] Research focusing on M&MM characteristics and the implementation of actions for improvement shows that the depth of analysis of the AE, including a focused discussion, is a key issue for effectiveness. [42] Although this may indicate that the discussion and analysis of the AE in our M&MMs were thorough, we may still question whether and what professionals actually learned. What constitutes learning for professionals may impact how the tasks assigned to them are valued and addressed to in the future. Perhaps professionals required deeper learning to make the PDCA-cycle effective and to integrate learning whilst executing tasks in their professional way of working. This means that in addition to learning how to follow the PDCA-cycle and finalize tasks, professionals may also connect learning from the M&MM to a normative level of what constitutes good care and leadership. The PDSA-cycle may be more suited to facilitate learning while implementing tasks from the M&MM. [43] It is important to not only create functional meetings, but facilitate reflective practices that lead to transform current practice. [44] This may facilitate more diverse discussions about the implications of areas for improvement for patient care and collaboration. When implementing this cyclical workflow in other settings this may be taken into account.

The success of a PDCA-cycle, which is a set framework, often lies in the adaptation to the local context and an iterative processes that may inform the next cycle. In our study several areas for improvement concerned topics discussed in previous cycles, such as wound infections. Therefore, several cycles iteratively informed other cycles and enabled one of the key features of a PDCA-cycle which is documentation. [20] Professionals in our study appreciated documentation, because it showed the steps that were taken to improve clinical practice and reached the goal of the M&MM which is learning from AEs. The reminders and repetitive contact may have stimulated professionals to act differently during implementation of the cyclic workflow. Even though literature shows that repetition may establish new habits, continuous reflection is preferred within a learning environment. [45, 46] The study approach of participatory action research (PAR) revealed that reflection, facilitated during interviews, was important for professionals to describe why their task was important and that they wanted to improve the quality of care. The time reserved to reflect on the actions in each of the following M&MMs contributed to the learning environment as well. However, as indicated in our study we advise to formulate tasks clearly and SMART (Specific, Measurable, Achievable, Realistic, Time-related). Moreover, it is important to additionally integrate new habits of reflection in the work setting outside of the M&MM. [36, 43].

The five factors that resulted from the research show that the social environment can positively or negatively stimulate behavior during the implementation of the cyclic workflow. A sense of commitment and support from the environment seemed to facilitate the successful execution of tasks. The tools based on the PDCA-cycle supported professionals by offering clarity, structure and a possibility to check tasks in the following meeting. We found that organizational culture seemed to have a strong influence on the successful implementation of our improvement strategy. Culture is a complex construct and general strategies to change and improve healthcare culture are lacking. [47,48] However, our study showed that commitment of professionals is an important factor while improving the follow-up of areas for improvement. This translates to a motivated professional which in effect can support changes in healthcare culture. In our study an external person stimulated professionals to follow-up on tasks. Although this may be related to a busy schedule of professionals that required reminders, it may also have been a way to motivate professionals to take on tasks. [49] This shows that there is a role for management or professionals in leading positions to facilitate a healthcare culture where attendees of the M&MM are motivated to implement tasks. Moreover, management may also take on facilitating a strong organizational structure around M&MMs that includes coordinating team members to improve the M&MM with a cyclical workflow. Overall, the implementation of a cyclic workflow required a committed professional to use the tools at the M&MM and to implement their assigned task.

Organizational commitment is defined as a force that ties an individual to work towards relevant targets. [50] When a group of professionals (employees, managers) experience commitment to an improvement strategy it may play a role in the successful implementation as it reduces resistance to organizational change. [51] Organizations with committed employees are more effective. When organizations wish to increase a sense of commitment in professionals it is important to create an environment that stimulates behavior to work on specific tasks and facilitate participation in teams. [52–54] A sense of participating in a team will be more clearly felt when attendees speak openly and freely without shame or blame during the M&MM. This can support the commitment of attendees in taking on tasks as a team effort. Although in some countries there might be a fear of legal or negative repercussions when AEs are openly discussed, M&MMs with patient participation at our department did not lead to more complaints or any form of litigation by patients. [55]

A strength of this study is the PAR approach that stimulated interaction with the researcher and the participants during the implementation of the cyclic workflow. This resulted in detecting and implementing necessary adjustments of the M&MM. The multidisciplinary research team provided diverse input during data collection and analysis. A comparative analysis of the follow-up of areas for improvement prior to the improvement strategies was not available. Therefore, this study could only establish the effectivity of the current improvement strategy based on the extent to which professionals followed-up on the PDCA-cycle and implemented the tasks. This research faced a limited and context specific setting in which the research is conducted. A possible bias is that the executive research was familiar with several professionals who received a task. Although putting an external person in charge of sending reminders may be a limitation to the sustainability in the current study context, we suggest that a coordinator who supports in the organization of M&MMs may function in this role external to the core team of professionals. Future research may include a comparative analysis to show the success rate of the implementation of learning points from the M&MM. Future research on success factors of implementing learning points from the M&MM may also benefit from using theoretical frameworks such as the Consolidated Framework for Implementation Research (CFIR) and the Theoretical Domains Framework (TDF) to investigate behavior change during implementation. [56, 57]

## Conclusion

In conclusion, an improvement strategy with a cyclic workflow and regular communication supports professionals at the M&MM to actively engage with their tasks and eventually improve clinical practice. It remains important to motivate professionals by putting an external person in charge of sending reminders, creating the right (learning) environment in- and outside of the M&MM to change behavior and sustain the uptake of the tools.

## Data Availability

The datasets used and/or analyzed during the current study are available from the corresponding author on reasonable request.
